# Translation and Cultural Adaptation of Tools to Assess Diverse Asian American and Asian Canadian Populations: The Asian Cohort for Alzheimer’s Disease Study

**DOI:** 10.1002/alz.092279

**Published:** 2025-01-09

**Authors:** Haeok Lee, Marian Tzuang, Tiffany W Chow, Younhee Kang, Boon Lead Tee, Clara Li, Pei‐Chuan Ho, Yian Gu, Anna T. Lu, Yun‐Beom Choi, Gyungah R Jun, Li‐San Wang, Haung (Ho) Yu, Van Ta Park, The Asian Cohort for Alzheimer’s Disease Study

**Affiliations:** ^1^ New York University, Meyers College of Nursing, New York, NY USA; ^2^ University of California San Francisco School of Nursing, San Francisco, CA USA; ^3^ University of Pennsylvania, Philadelphia, PA USA; ^4^ Ewha Womans University, Seoul Korea, Republic of (South); ^5^ Memory and Aging Center, UCSF Weill Institute for Neurosciences, University of California, San Francisco, San Francisco, CA USA; ^6^ Department of Psychiatry, Icahn School of Medicine at Mount Sinai, New York, NY USA; ^7^ University of Pennsylvania Perelman School of Medicine, Philadelphia, PA USA; ^8^ Columbia University Irving Medical Center, New York, NY USA; ^9^ University of California San Diego, Alzheimer’s Disease Cooperative Study (ADCS), La Jolla, CA USA; ^10^ Englewood Health, Englewood, NJ USA; ^11^ Rutgers New Jersey Medical School, East Orange, NJ USA; ^12^ Department of Medicine (Biomedical Genetics), Boston University Chobanian & Avedisian School of Medicine, Boston, MA USA; ^13^ Penn Neurodegeneration Genomics Center, Dept of Pathology and Laboratory Medicine, University of Pennsylvania, Philadelphia, PA USA; ^14^ University of Toronto, Pharmacology and Toxicology, Toronto, ON Canada

## Abstract

**Background:**

Socio‐cultural and language‐appropriate study materials and instruments are critical for accurate assessment of cognitive function in people from diverse backgrounds. Most research uses cognitive tests based on Western, industrialized, English‐speaking cultures and may not reflect global experiences. The purpose of this study was to describe the translations of study materials and cultural adaptations that were developed for the Asian Cohort for Alzheimer’s Disease (ACAD).

**Methods:**

Multi‐lingual researchers, clinicians, and community leaders with extensive practical translation experience created materials and instruments in accordance with World Health Organization (WHO) guidelines, using a translation process that consisted of preparing materials, translating materials, conducting a committee review, conducting a pretest (as appropriate), and conducting necessary revisions. The ACAD study population speaks Chinese (Simplified and Traditional), Korean, or Vietnamese. The majority of instruments in the protocol harmonize with the National Alzheimer’s Coordinating Center purposefully, warranting both literal and conceptually cultural‐appropriate translations for our older Asian American and Asian Canadian participants.

**Results:**

We have developed Asian language versions of the ACAD informed consent, data collection packet, and community outreach materials for all our target groups. Many cognitive tools had been previously translated into Asian languages and made minor modifications. Some items required cultural adaptations to reflect the socio‐cultural background of the targeted Asian languages, e.g., the types of physical activities or dietary choices surveyed.

**Conclusion:**

This multi‐stage translation process accounts for the distinctive socio‐cultural and language backgrounds of each Asian population. Alzheimer’s disease and related dementias researchers interested in engaging with these populations may apply this translation process to reduce health disparities in these underrepresented populations. To ensure fidelity across different languages, ACAD will continue to engage in this comprehensive translation process with our diverse community stakeholders.

